# Ghrelin modulates testicular damage in a cryptorchid mouse model

**DOI:** 10.1371/journal.pone.0177995

**Published:** 2017-05-18

**Authors:** Enrica Bianchi, Kim Boekelheide, Mark Sigman, Susan J. Hall, Kathleen Hwang

**Affiliations:** 1 Division of Urology, Rhode Island Hospital, Providence, RI, United States of America; 2 Department of Pathology and Laboratory Medicine, Brown University, Providence, RI, United States of America; Centre de Recherche en Cancerologie de Lyon, FRANCE

## Abstract

Cryptorchidism or undescended testis (UDT) is a common congenital abnormality associated with increased risk for developing male infertility and testicular cancer. This study elucidated the effects of endogenous ghrelin or growth hormone secretagogue receptor (GHSR) deletion on mouse reproductive performance and evaluated the ability of ghrelin to prevent testicular damage in a surgical cryptorchid mouse model. Reciprocal matings with heterozygous/homozygous ghrelin and GHSR knockout mice were performed. Litter size and germ cell apoptosis were recorded and testicular histological evaluations were performed. Wild type and GHSR knockout adult mice were subjected to creation of unilateral surgical cryptorchidism that is a model of heat-induced germ cell death. All mice were randomly separated into two groups: treatment with ghrelin or with saline. To assess testicular damage, the following endpoints were evaluated: testis weight, seminiferous tubule diameter, percentage of seminiferous tubules with spermatids and with multinucleated giant cells. Our findings indicated that endogenous ghrelin deletion altered male fertility. Moreover, ghrelin treatment ameliorated the testicular weight changes caused by surgically induced cryptorchidism. Testicular histopathology revealed a significant preservation of spermatogenesis and seminiferous tubule diameter in the ghrelin-treated cryptorchid testes of GHSR KO mice, suggesting that this protective effect of ghrelin was mediated by an unknown mechanism. In conclusion, ghrelin therapy could be useful to suppress testicular damage induced by hyperthermia, and future investigations will focus on the underlying mechanisms by which ghrelin mitigates testicular damage.

## Introduction

Cryptorchidism, also known as undescended testis (UDT), is one of the most common abnormalities of male sexual development characterized by failure of the testis to descend into the scrotum where temperature is optimal for spermatogenesis [1]. Higher abdominal temperatures alter normal testicular function and cause the formation of multinucleated giant cells and germ cell loss from the seminiferous epithelium with subsequent arrest of spermatogenesis [2]. The mechanism of heat-induced cell death is mediated by apoptosis and not necrosis, in which primary spermatocytes and spermatids are the most susceptible cells [3–5], while Sertoli cells, Leydig cells and androgen secretion do not appear to be directly affected [6]. Previous studies suggest that the mechanism of elevated temperature inducing cell death may involve reactive oxygen species (ROS) [7], nitric oxide synthase (NOS) [8], translocation of pro-apoptotic factor Bax from the cytoplasm to a perinuclear position, and release of cytochrome c from the mitochondria [4, 9–11].

Ghrelin, a n-octanoylated 28 amino acid peptide, has been identified as the endogenous ligand for a specific G-protein-coupled receptor called growth hormone secretagogue receptor (GHSR) [12, 13]. Two GHSR subtypes have been described: the functionally active GHSR type 1a and the truncated GHSR type 1b, generated by alternative splicing of the same gene [14]. Ghrelin is a multifunctional peptide that acts centrally to regulate growth hormone secretion, food intake and energy balance. Recently, our group demonstrated the anti-inflammatory and anti-fibrotic effects of ghrelin in a surgical mouse model of adhesion induction mediated via the GHSR-1a signaling pathway [15, 16]. Previous studies revealed the presence of endogenous ghrelin and its receptor in many peripheral tissues including the testis [17, 18], suggesting a role for this endogenous peptide in the direct control of male gonadal function [19]. Ghrelin is mainly expressed in the interstitial Leydig cells of human and rat testes and within the seminiferous tubules in Sertoli cells of human testes. In addition, the GHSR receptor has been detected in rat and human Leydig and Sertoli cells [20–22].

The direct action of ghrelin in inhibiting apoptosis has been demonstrated in multiple cell types, including cardiomyocytes [23, 24], pituitary cells [25], pancreatic β cells [26, 27] and adipocytes [28], while its pro-apoptotic effect has been detected in other cell types, such as ovarian granulosa [29] and endothelial cells [30]. Recent findings demonstrated that ghrelin is an endogenous antioxidant and functions as a free radical scavenger [31]. It has been demonstrated that ghrelin inhibits apoptosis by down regulation of Bax, preventing cytochrome c release [32], and inhibition of reactive oxygen species (ROS) formation, increasing antioxidant enzyme activities and reducing lipid peroxidation [33, 34] in rat testes. Although these protective effects of ghrelin in preventing testicular damage have been previously observed, the receptor and molecular mechanisms involved in mediating these effects remain unknown.

The present study was designed to test whether ghrelin and its functional receptor GHSR1a are key mediators in mouse spermatogenesis to improve the understanding of mouse reproductive biology. We demonstrated that ghrelin knockout (KO) mice have a significant increase in germ cell apoptosis and a significant decrease in litter size for breeding pairs that included ghrelin KO males. While we hypothesized that the GHSR pathway, following ghrelin administration, was involved in preventing surgically-induced testicular damage we found that this protective response was also detected in GHSR KO mice, indicating that the ghrelin induced antioxidant effect may be mediated by a second receptor that has not been identified.

## Materials and methods

### Animals

Male wild type C57BL/6 mice (n = 80), 50–55 days old, were purchased from Charles River Laboratories (Wilmington, MA). All mice were allowed free access to Purina Rodent Chow 5010 (Farmer’s Exchange, Framingham, MA) and water *ad libitum*. Mice were housed in the Brown University Animal Care Facility and kept in a continuous 12 hrs alternating light-dark cycle with controlled temperature (25–28°C) and humidity (30–70%). All investigations were conducted in accordance with The Guide for the Care and Use of Laboratory Animals and were approved by the Brown University Institutional Animal Care and Use Committee (IACUC protocol number: 1412000111).

### Ghrelin KO and GHSR KO mice

Growth hormone secretagogue receptor knockout (GHSR KO) mice and ghrelin knockout (ghrelin KO) mice, C57BL/6 mice with a deletion of the growth hormone secretagogue receptor (ghsr^-/-^) or ghrelin (ghrelin^-/-^), were developed at Baylor College of Medicine (Houston, Texas) [35, 36]. GHSR KO and ghrelin KO mice were backcrossed at least 10 generations to C57BL/6 mice to create isogenic lines. The GHSR and ghrelin knockout mice were bred in-house using the following mating scheme:

1. GHSR knockout transgenic mouse line (female GHSR +/- x male GHSR +/-, female GHSR +/- x male GHSR -/-, female GHSR -/- x male GHSR +/-, female GHSR -/- x male GHSR -/-).
2. ghrelin knockout transgenic mouse line (female ghrelin +/- x male ghrelin +/-, female ghrelin +/- x male ghrelin -/-, female ghrelin -/- x male ghrelin +/-, female ghrelin -/- x male ghrelin -/-).

### Matings

Females and males of heterozygous (+/-) or homozygous (-/-) ghrelin or/and GHSR knockout mice were paired and the newborn pups were counted for every mating pair. Pups were tail clipped before 21 days of age for genotyping in accordance with Brown’s “Mouse Tail Biopsy Policy” approved by the local ethics committee. Testes of adult mice were collected, weighed and embedded in paraffin for histological analysis.

### Histology

To examine the morphological appearance of seminiferous tubules of both the transgenic mouse lines, testes were collected and fixed overnight in Bouin’s solution, dehydrated in a descending series of ethanol washes and embedded in paraffin. 5 μm sections were stained with hematoxylin and eosin (H&E), cleared with isopropanol and Citrisolv (Thermo Fisher Scientific, Waltham, MA), mounted using Cytoseal-60 (Thermo Fisher Scientific) and examined under a light microscope.

### Assessment of apoptosis by TUNEL assay

Germ cell apoptosis was detected in 5 μm paraffin sections of wild type (WT), ghrelin knockout (ghrelin KO) and GHSR knockout (GHSR KO) mice testes using the Apoptag Peroxidase In Situ Apoptosis Detection Kit (TUNEL kit, EMD Millipore, Billerica, MA) according to the manufacturer’s instructions. Slides were scanned into an Aperio ScanScope CS microscope (Aperio Technologies, Vista, CA). TUNEL-positive cells and seminiferous tubule diameter were measured using Leica ImageScope software (Leica Biosystems Inc, Buffalo Grove, IL). TUNEL positive cells were counted in an average of 152 ± 24 (wild type mice, n = 4), 169 ± 33 (ghrelin KO mice, n = 4) and 148 ± 22 (GHSR KO mice, n = 6) seminiferous tubules for each cross section having a ratio major axis/minor axis < 1.5. The data were expressed as the percentage of round seminiferous tubules with more than 3 TUNEL-positive cells.

### Chemicals

1 mg rat lyophilized acylated ghrelin (Tocris Bioscience, Bristol, UK) was dissolved in 1 ml of sterile saline (Baxter Healthcare Corporation, IL).

### Experimental groups

A total of 80 male C57BL/6 wild type mice and 20 male GHSR KO, 50–55 days of age and weighing between 19–21 g, underwent a midline laparotomy to establish a surgical unilateral cryptorchid model. They were randomly separated into two groups to receive saline as control group and ghrelin as treatment group. In the control group, C57BL/6 wild type and GHSR KO mice were injected intraperitoneally twice daily with 0.1 mL saline, whereas in the treatment group, animals received intraperitoneal injections of 0.16 mg/kg ghrelin diluted in 0.1 mL saline twice daily for 1 day post-surgery (saline treated-wild-type mice = 10 and ghrelin treated-wild-type mice = 10), 4 days post-surgery (saline treated-wild-type mice = 10 and ghrelin treated-wild-type mice = 10) or 20 days post-surgery (saline treated-wild-type mice = 20, ghrelin treated-wild-type mice = 20, saline-treated GHSR KO mice = 10, ghrelin-treated GHSR KO mice = 10). Mice were euthanized by isoflurane overdose according to IACUC-approved protocols.

### Surgical procedures

Surgical procedure for induction of unilateral cryptorchidism was performed. Anesthesia was induced with isoflurane (Baxter Healthcare Corp) gas and maintained with 2–3% isoflurane via a nose cone throughout the entire sterile surgical procedure. After anesthesia, the surgical area was shaved and prepared by povidone iodine solution. A midline abdominal incision was made, and the right testis was manipulated into the abdomen and sutured to the abdominal wall by a 6–0 gut absorbable suture (Ethicon Inc, Somerville, NJ). For each animal the right testis was treated as the experimental organ, whereas the left testis remained completely untouched throughout the procedure and acted as a control. After the laparotomy incision was closed into layers with a 5–0 Vicryl suture (Ethicon Inc, Somerville, NJ).

### Sampling and tissue preparation

After the mice were euthanized at 1, 4 and 20 days post-surgery, cryptorchid and normal testes were collected and weighed. The testes were cut in half. One half was fixed in Bouin’s solution to be embedded in glycol methacrylate (Technovit 7100, Heraeus Kulzer GmBH, Germany) for histological analysis and the other half was snap-frozen in liquid nitrogen for glutathione (GSH) content analysis. The frozen half of the testes were thawed and manually homogenized in cold phosphate-buffered saline pH 7.4 (PBS) and 2mM EDTA (ice cold), 1 ml of PBS/EDTA per 10 mg of tissue. Insoluble material was removed by centrifugation at 4°C, 10 minutes, 14,000 g, and the supernatant was collected and recovered for GSH-Glo Glutathione Assay (Promega, Madison, WI).

### Morphology quantification

Cross-sections from the middle of each testis embedded in glycol methacrylated (Technovit 7100, Heraeus Kulzer GmBH, Germany) were stained with periodic acid Schiff’s reagent and hematoxylin (PASH). Blinded slides were examined on an Olympus BH-2 light microscope (Waltham, MA) for quality of histology and scanned into an Aperio ScanScope CS microscope (Aperio Technologies, Vista, CA). Histological endpoints were measured using Leica ImageScope software. The following endpoints were used to assess testicular damage: testicular weights, seminiferous tubule diameter, percentage of tubules with spermatids, and percentage of tubules with giant cells.

### Total GSH content

Total amount of glutathione (GSH) was evaluated by GSH-Glo Glutathione Assay kit (Promega, Madison, WI) according to the manufacturer’s instructions. Normal cells possess a well-developed biochemical defense system, comprising free radical scavengers such as glutathione (GSH), vitamin C, vitamin E, glutathione peroxidase (GPx), superoxide dismutase activity (SOD) and tissue catalase activity (CAT) [37]. In addition, a decrease in intracellular GSH or an increase in glutathione disulphide (GSSG) constitutes a trigger for apoptosis [38, 39]. GSH-Glo Glutathione Assay is a luminescent-based assay for the detection and quantification of glutathione (GSH) in biological samples using a Microplate Reader Spectra Max M5. GSH content was expressed as μM per 10 mg of tissue.

### Statistical analysis

Statistical analysis was performed using GraphPad Prism software (La Jolla, CA). Multiple comparisons between breeding pairs of ghrelin and GHSR heterozygous (+/-) and knockout (-/-) knockout mice or multiple were obtained using a one-way analysis of variance (ANOVA) with Bonferroni post-hoc test. Multiple comparison between saline- and ghrelin-treated wild type and GHSR KO normal and cryptorchid testis were conducted using a one-way analysis of variance (ANOVA) with Fisher’s LSD test. One-way analysis of variance (ANOVA) with Dunnett’s post-hoc test was performed to compare the experimental groups to the normal wild type samples. Finally, two-way analysis of variance (ANOVA) with Fisher’s LSD test was used to compare the ratio of cryptorchid to scrotal testis between ghrelin- and saline-treated wild type mice at 1, 4 and 20 days post-surgery. All data were presented as mean ± standard error of the mean (SEM). Values were considered to be significant at p < 0.05.

## Results

The mean litter size of wild type C57BL/6 mice has been previously reported as approximately 7 pups per litter [40]. No differences were detected between the mean litter size of heterozygous ghrelin knockout (6.625 ± 0.831, n = 16) and heterozygous GHSR knockout (7.455 ± 0.692, n = 11) mice matings (data not shown).

### Reproductive performance of ghrelin KO mice

**Fig 1:**
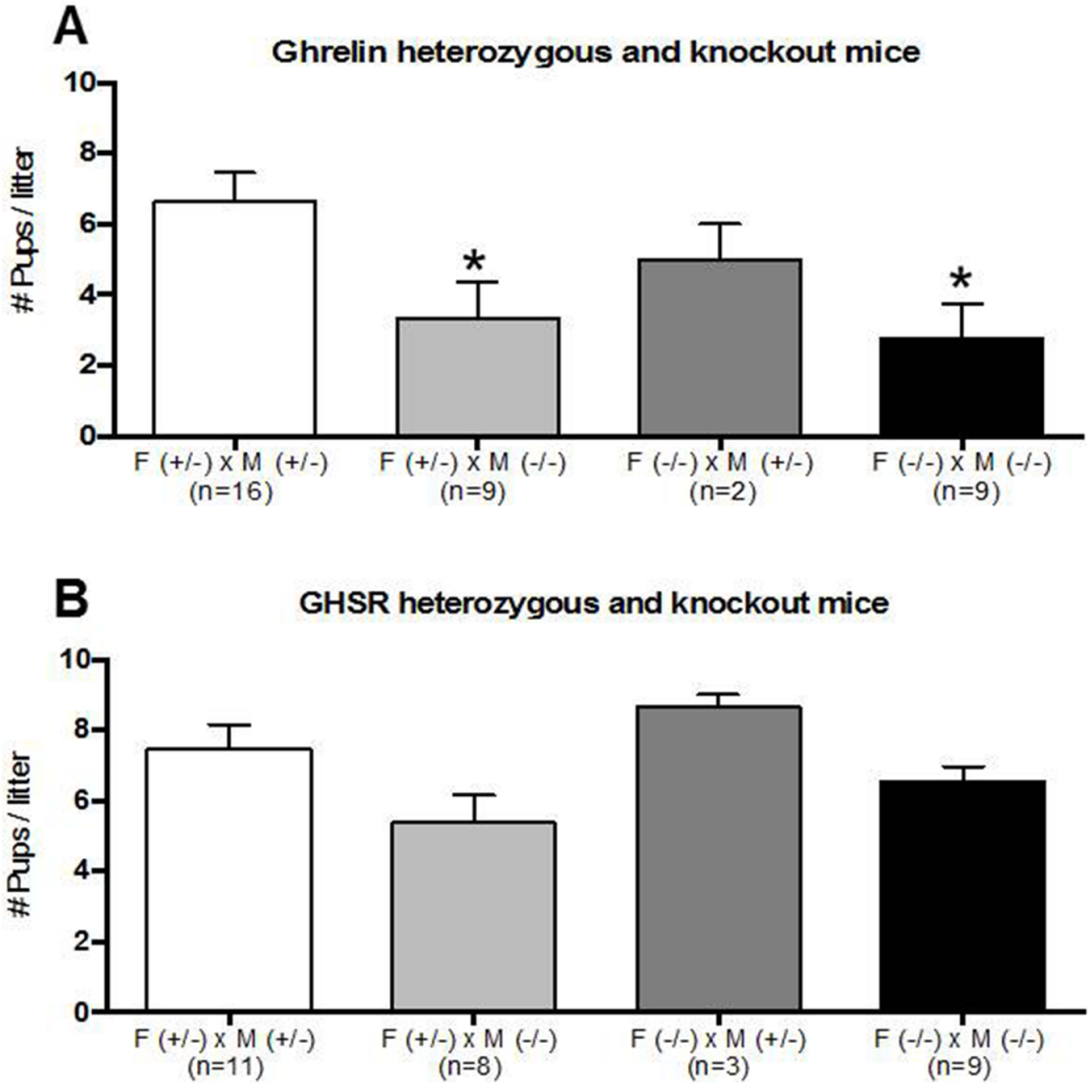
Evaluation of the reproductive performance of ghrelin KO and GHSR KO mice. A) Breeding pairs of ghrelin heterozygous (+/-) and knockout (-/-) mice were co-housed for 10 days and the number of pups per litter was determined. Breeding pairs that included ghrelin KO males showed a significant decrease in the number of pups. B) Breeding pairs of GHSR heterozygous (+/-) and knockout (-/-) mice were co-housed for 10 days and the number of pups per litter was determined. No significant differences were detected among the different mating groups. Only breeding pairs with mice between 2 and 8 months old were included. Data were analyzed by one-way ANOVA multiple comparisons with Bonferroni's test and expressed as mean ± SEM (* p<0.05), and compared to the control (+/-) x (+/-) matings.

The impact of ghrelin or GHSR deletion on reproductive success was assessed by analyzing litter size after mating heterozygous and/or homozygous knockout mice. Four groups of breeding pairs for the ghrelin knockout transgenic mouse line with the following genotypes were housed together for 10 days: female ghrelin +/- and male ghrelin +/- (group one, n = 16), female ghrelin +/- and male ghrelin -/- (group two, n = 9), female ghrelin -/- and male ghrelin +/- (group three, n = 2), female ghrelin -/- and male ghrelin -/- (group four, n = 9). No litters were born from 3 female ghrelin +/- X male ghrelin -/- pairs, 4 female ghrelin -/- X male ghrelin -/- pairs, 1 female ghrelin +/- X male ghrelin +/- pair and 1 female ghrelin -/- X male ghrelin +/- pair. Female ghrelin +/- and male ghrelin -/- breeding pairs (3.333 ± 1.014, n = 9 p-value = 0.048) and female ghrelin -/- and male ghrelin -/- breeding pairs (2.778 ± 0.969, n = 9 p-value = 0.017) showed a significant decrease in the number of pups per litter compared to the female ghrelin +/- and male ghrelin +/- breeding pairs (6.625 ± 0.831, n = 16) designed as the control group. No significant differences were detected for female ghrelin -/- and male ghrelin +/- breeding pairs in the number of pups per litter compared to the control group (Fig 1A).

### Reproductive performance of GHSR null mice

Four groups of breeding pairs for the GHSR knockout transgenic mouse line with the following genotypes were housed together for 10 days: Female GHSR +/- and male GHSR +/- (group one, n = 11), female GHSR +/- and GHSR -/- (group two, n = 8), female GHSR -/- and male GHSR +/- (group three, n = 3), female GHSR -/- and male GHSR -/- (group four, n = 9). No litters were born from 2 female GHSR +/- X male GHSR -/- pairs and 2 female GHSR -/- X male GHSR -/- pairs. No significant differences were detected in the number of pups per litter compared to the female GHSR +/- and male GHSR +/- breeding pairs designed as control group (Fig 1B).

### Ghrelin KO and GHSR KO mice: Testicular weight and histology

**Fig 2:**
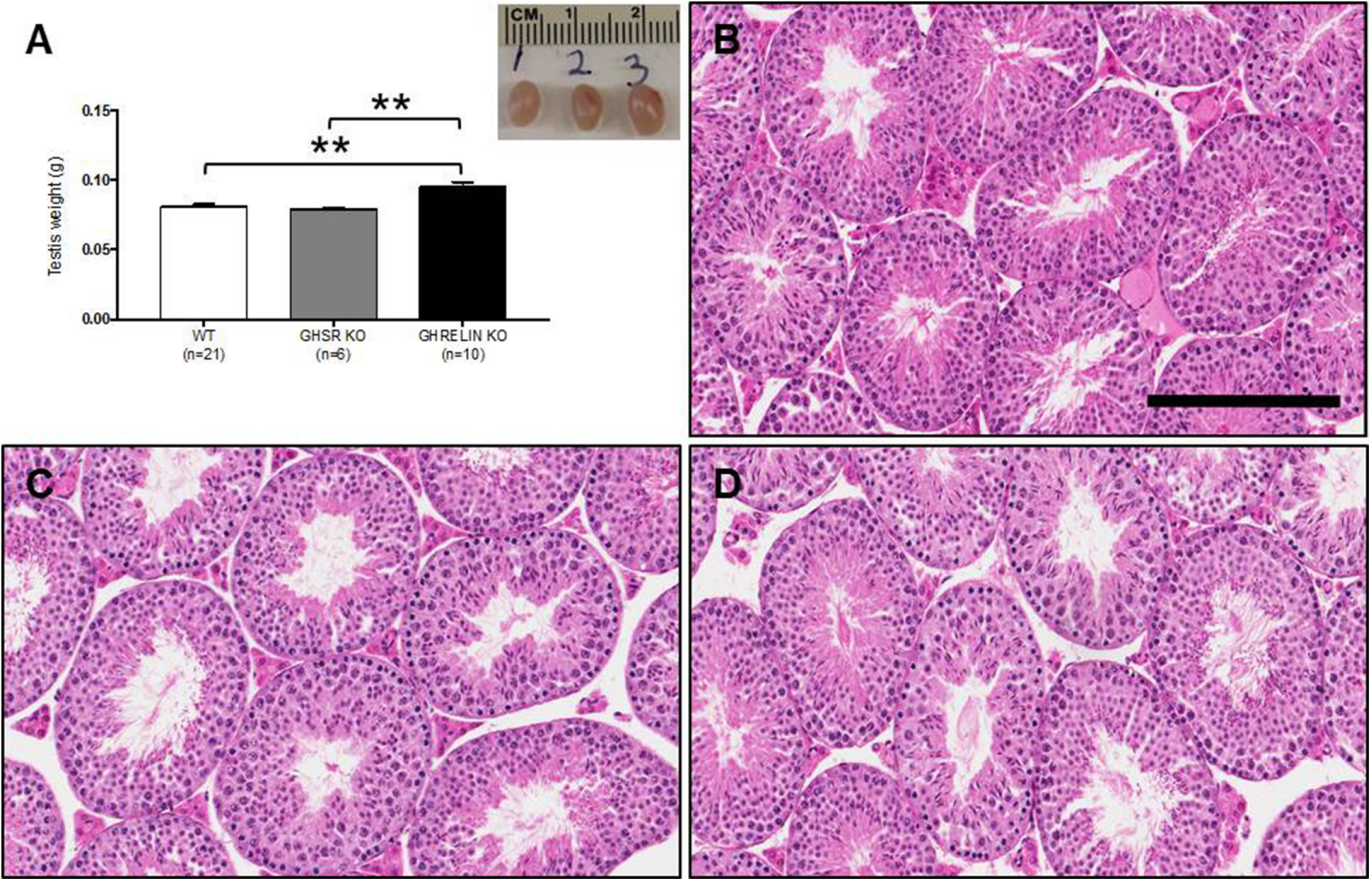
Increased testicular weight in ghrelin KO mice. A) Testicular weights and picture of WT, GHSR KO and Ghrelin KO testes. The right testis weight was significantly increased in ghrelin KO mice compared to the WT and GHSR KO mice (80 day old). No significant differences were detected in testis weight between WT mice and GHSR KO mice. Photomicrographs of testis cross-sections from 80 day old WT mice (B), ghrelin KO mice (C) and GHSR KO mice (D). Scale bar = 200 μm. Data were analyzed by one-way ANOVA multiple comparisons Bonferroni's test and expressed as mean ± SEM (** p<0.01).

The right testis weight of ghrelin KO, GHSR KO and wild type male mice was assessed. The testicular weights were significantly increased (p-value = 0.002, p-value = 0.006) in ghrelin KO mice (0.095 ± 0.004, n = 10) compared to the wild-type (0.081 ± 0.002, n = 21) and GHSR KO mice (0.079± 0.002, n = 6). No significant differences in testis weight were detected between GHSR KO mice and wild-type mice (Fig 2A).

**Fig 3:**
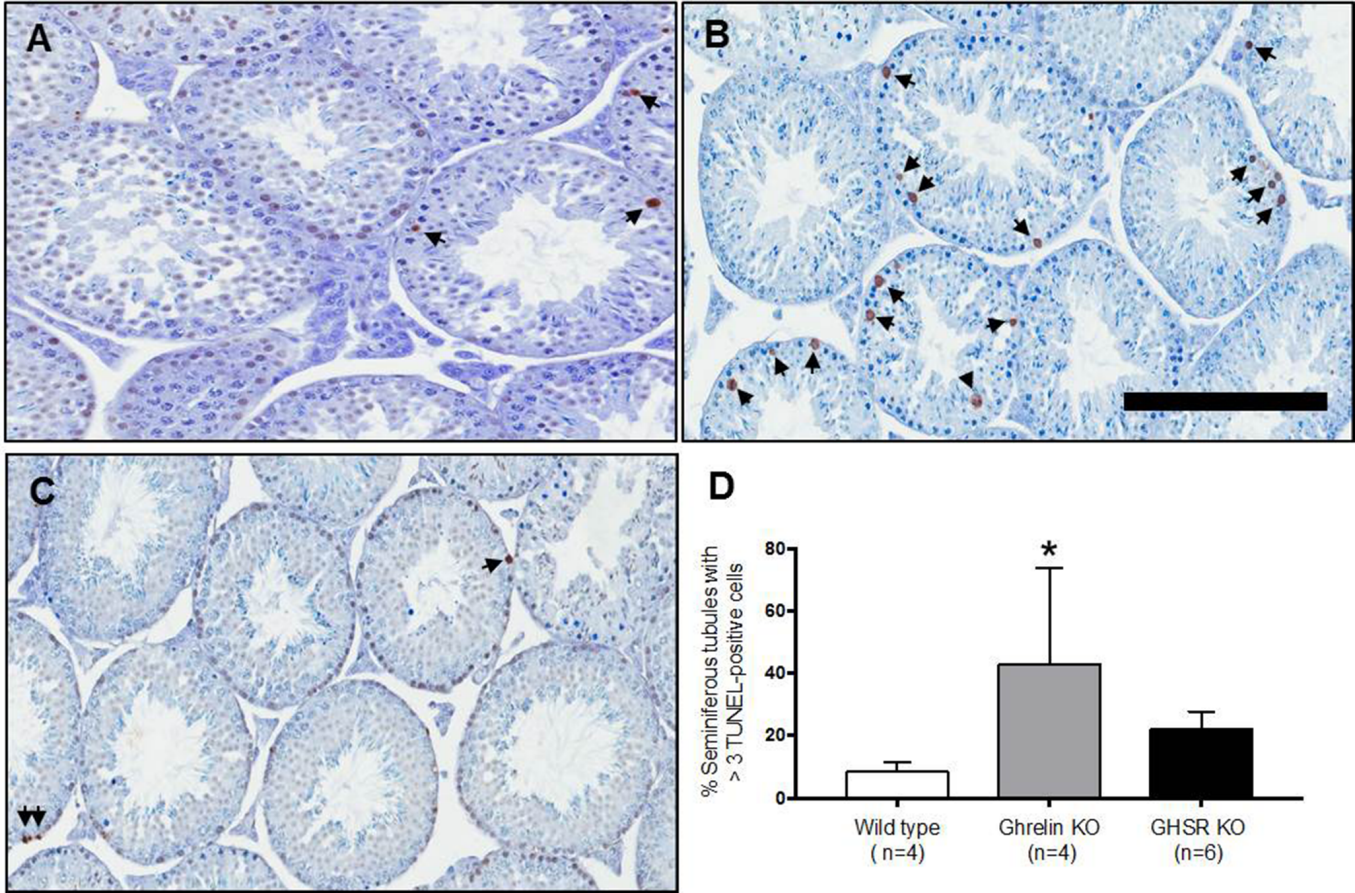
Increased germ cell apoptosis in ghrelin KO mice. Apoptotic germ cells were detected by TUNEL analysis in wild type (A), ghrelin KO (B) and GHSR KO (C) mice. The arrows indicate apoptotic germ cells. Scale bar = 200μm. D) Quantitation of TUNEL-positive cells in wild type, ghrelin KO and GHSR KO mice testes. The data were expressed as the percentage of round seminiferous tubules (ratio major axis/minor axis < 1.5) with more than 3 TUNEL-positive cells. Ghrelin KO mice showed a significant increase in the percentage of seminiferous tubules with >3 TUNEL-positive cells compared to wild type mice. Data were analyzed by one-way ANOVA multiple comparisons Bonferroni's test and expressed as mean ± SEM (* p<0.05).

Histological cross sections of wild type (Fig 2B), ghrelin KO (Fig 2C) and GHSR KO (Fig 2D) mice testes were examined. Evaluation of testis histopathology was conducted by an expert pathologist who was blinded to the sample groups. No histological abnormality was detected in any of the testes of these transgenic mouse lines. Apoptotic germ cells were measured by TUNEL staining in testes of wild type (Fig 3A), ghrelin KO (Fig 3B) and GHSR KO (Fig 3C) mice. Quantification of TUNEL-positive cells in cross sections of ghrelin KO mice testes (42.704% ± 15.582, n = 4 p = 0.029) showed a significant increase in the percentage of seminiferous tubules with more than 3 TUNEL-stained cells compared to the control wild type mice (8.372% ± 1.569, n = 4). No difference was detected in the percentage of seminiferous tubules with more than 3 TUNEL-positive cells in GHSR KO mice (21.916% ± 2.352, n = 6) compared to the control group (Fig 3D).

### Ghrelin treatment ameliorates testicular weight changes caused by heat-injury

**Fig 4:**
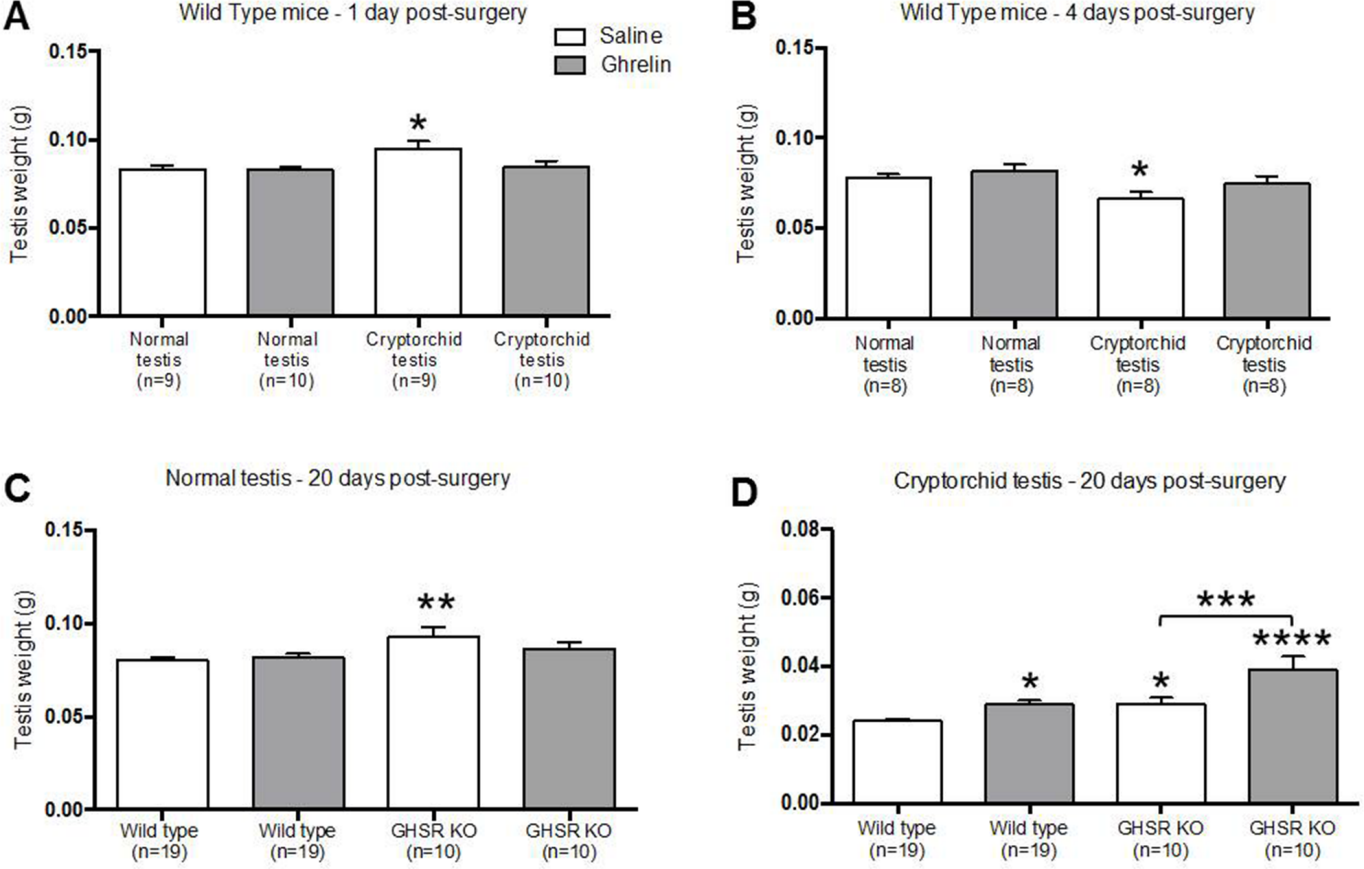
Ghrelin prevented testicular weight change in wild type and GHSR KO cryptorchid mice. At 1 day-post surgery, testis weight was significantly increased in saline-treated cryptorchid testes compared to saline-treated normal testes. This initial testicular swelling was prevented by ghrelin treatment (A). At 4 days post-surgery, testis weight was significantly decreased in saline-treated cryptorchid testes compared to saline-treated normal testes, and this effect was ameliorated by ghrelin treatment (B). The normal testis weight was significantly increased in saline-treated GHSR KO mice compared to the saline-treated wild type mice (C). Ghrelin administration significantly increased testis weight compared to the respective controls in both wild type and GHSR KO cryptorchid mice at 20 days post-surgery (D). Data were analyzed by one-way ANOVA multiple comparisons with Fisher’s LSD test and expressed as mean ± SEM (* p<0.05, ** p<0.01, *** p<0.001, **** p<0.0001).

Testes of the mature male mice surgically confined into the abdominal cavity showed a significant increase in the testicular weight at 1 day post-surgery (Fig 4A) (0.095 g ± 0.005, n = 9) compared to the normal testis (0.083 g ± 0.002, n = 9). This increase of testicular weight was followed by loss of testicular weight at 4 days post-surgery (Fig 4B) (0.066 g ± 0.004, n = 8) that was further exaggerated at 20 days after surgery (Fig 4D) (0.024 g ± 0.001, n = 19). Ghrelin treatment prevented changes in the weight of cryptorchid testes. Saline-treated cryptorchid wild-type mice at 1 day post-surgery (0.095 g ± 0.005, n = 9) showed a significant (p = 0.024) increase in testicular weight compared to the normal testes (0.083 g ± 0.002, n = 9). However, ghrelin-treated cryptorchid wild-type mice (0.085 g ± 0.003, n n = 9) exhibited a similar testicular weight of the normal testes (0.083 g ± 0.002, n = 9) (Fig 4A). At 4 days post-surgery saline-treated cryptorchid wild-type mice (0.066 g ± 0.004, n = 8) displayed a significant decrease (p = 0.267) in testicular weight compared to the normal testes (0.078 g ± 0.002, n = 8). Ghrelin treatment induced an increase in testicular weight (0.075 g ± 0.004, n = 8). No significant weight differences were detected between ghrelin-treated cryptorchid testis (0.075 g ± 0.004, n = 8) and normal testis (0.078 g ± 0.002, n = 8) at 4 days after surgery (Fig 4B). At 20 days post-surgery saline-treated GHSR KO normal testis (0.093 g ± 0.005, n = 10) were significantly (p = 0.0021) increased compared to wild type mice (0.080 g ± 0.002, n = 19). Ghrelin-treated GHSR KO normal testis (0.086 g ± 0.003, n = 10) showed a similar testicular weight of the wild type normal testis (Fig 4C). Ghrelin-treated wild-type cryptorchid mice (0.029 g ± 0.001, n = 19) showed significant (p<0.05) protection of testicular weight compared to the saline-treated wild-type cryptorchid mice (0.024 g ± 0.001, n = 20). In addition, cryptorchid testes of ghrelin-treated GHSR KO mice (0.039 g ± 0.004, n = 10) showed a significantly (p-value = 0.001) higher weight compared with the saline-treated cryptorchid GHSR KO mice testes (0.029 g ± 0.002, n = 10) (Fig 4D).

### Ghrelin protects against disruptions in spermatogenesis

**Fig 5:**
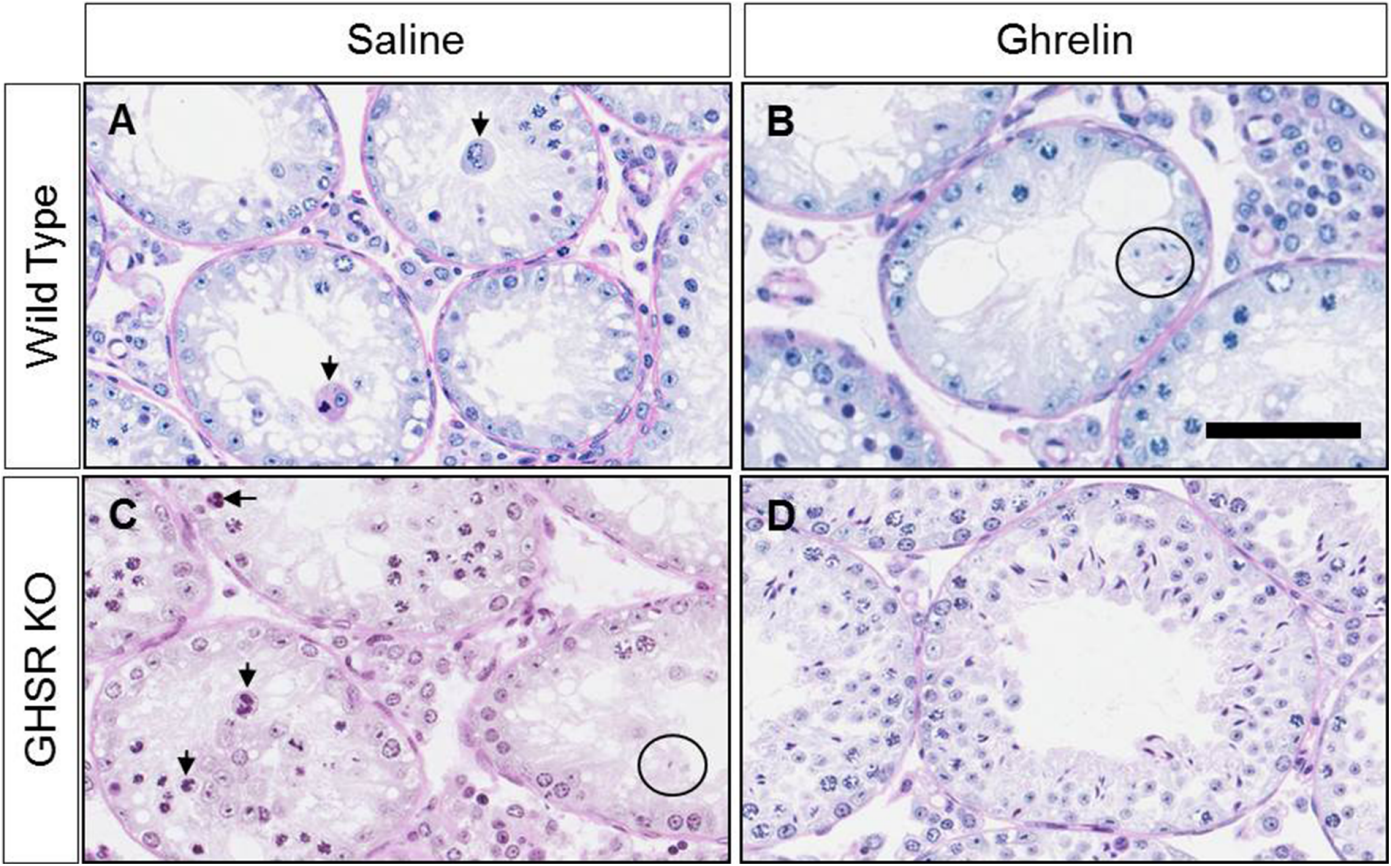
Histopathological analysis of ghrelin and saline-treated cryptorchid testes excised from wild type and GHSR KO mice at 20 days post-surgery. Cryptorchid testes from saline-treated wild type and GHSR KO mice showed degeneration of seminiferous tubules with evidence of degenerating multinucleated giant cells (arrow) at 20 days post-surgery (A, C). Ghrelin-exposed animals revealed an increase in the number of spermatids (circle) in the seminiferous tubules and a reduction in the number of multinucleated giant cells compared to the control mice (B,D). Bars = 200 μM; periodic acid Schiff and hematoxylin staining.

**Fig 6:**
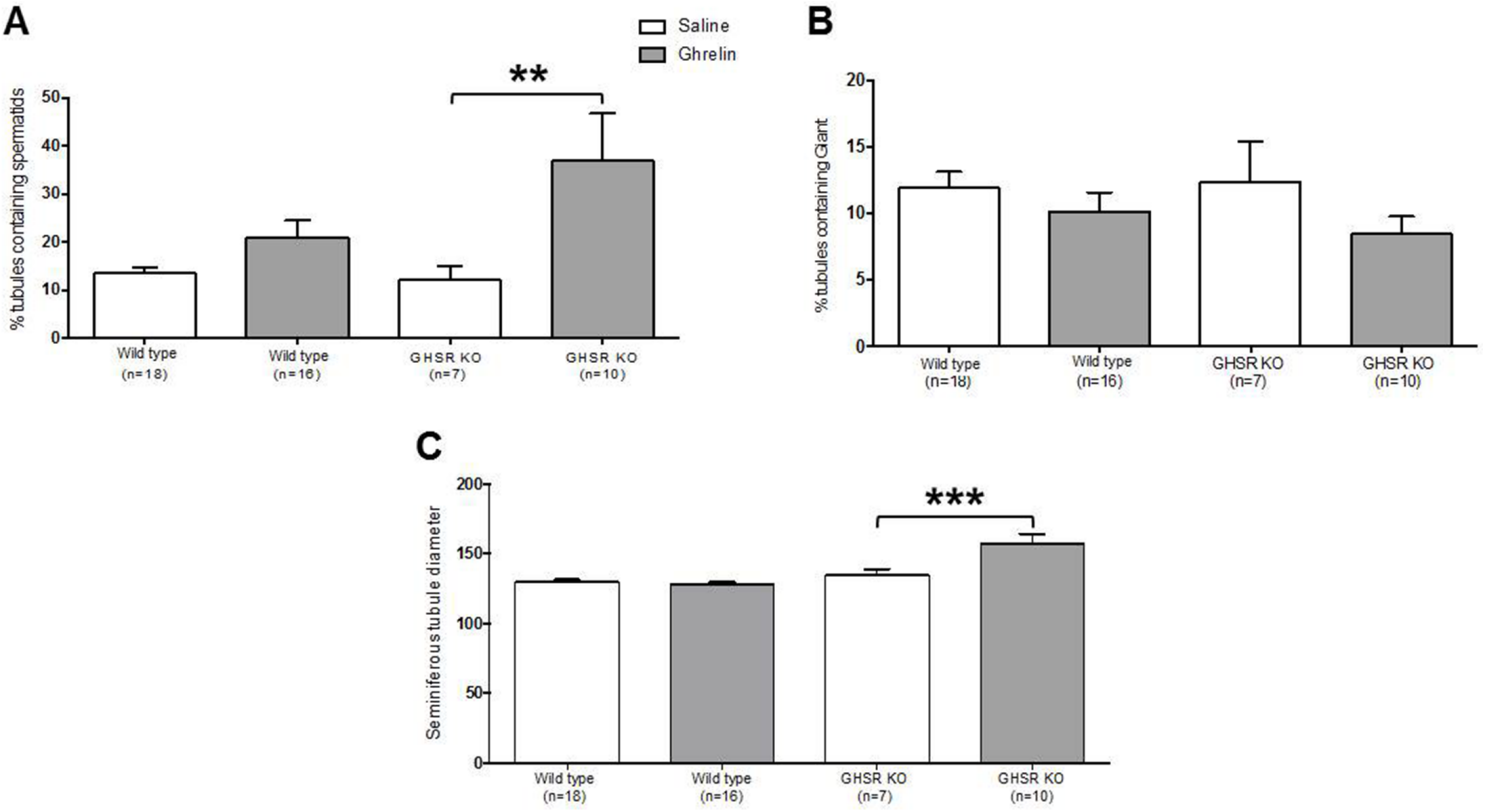
Quantification of spermatid-containing tubules, seminiferous tubule diameter and multinucleated giant cells (MnGC) in cryptorchid testes of wild type and GHSR KO mice. A) The number of tubules containing spermatids was significantly higher in cryptorchid testes of ghrelin-treated GHSR KO mice than in the control. B) No significant differences in the number of tubules containing MnGC were detected between ghrelin- and saline-treated cryptorchid testes. C) Ghrelin-treated GHSR KO mice showed significant increase in minor axis seminiferous tubule diameter compared to the control. Data were analyzed by one-way ANOVA multiple comparisons with Fisher’s LSD test and expressed as mean ± SEM (** p<0.01, *** p<0.001).

Severe time-dependent disorganization of the seminiferous tubules was observed in cryptorchid testes. Histopathological examinations showed deterioration in germinal epithelium and seminiferous tubules. At 20 days post-surgery all germinal elements of the tubules were lost with a single layer of Sertoli cells next to the basement membrane of the tubules (data not shown). Examination of Periodic Acid Schiff and Hematoxylin-stained sections of cryptorchid testes from wild-type and GHSR KO mice at 20 days post-surgery was performed. Saline-treated cryptorchid wild type and GHSR KO mice showed degeneration of seminiferous tubules with an accumulation of multinucleated giant cells at 20 days after surgery (Fig 5A and 5C). Multinucleated giant cells were less common in ghrelin-treated cryptorchid mice compared to the control animals (Fig 5). However, no significant differences were detected in the percentage of seminiferous tubules with multinucleated giant cells in ghrelin-treated mice compared to the control group (Fig 6B). The percentage of round seminiferous tubules containing spermatids was significantly higher (p-value = 0.003) in ghrelin-treated cryptorchid GHSR KO mice (36.984% ± 9.888, n = 10) compared to the saline-treated GSHR KO cryptorchid testes (12.091% ± 2.936, n = 7) (Fig 6A). No differences in the average seminiferous tubule diameter were detected in cryptorchid testes between ghrelin-treated (127.641 ± 1.645, n = 16) and saline-treated (129.383 ± 1.884, n = 18) wild-type mice. However, the average seminiferous tubule diameter was significantly increased (p-value = 0.0002) in ghrelin-treated GHSR KO mice (157.404 ± 6.493, n = 10) compared to the saline-treated GHSR KO group (134.599 ± 4.307, n = 7) (Fig 6C).

### Ghrelin treatment attenuates testicular loss of GSH content

**Fig 7:**
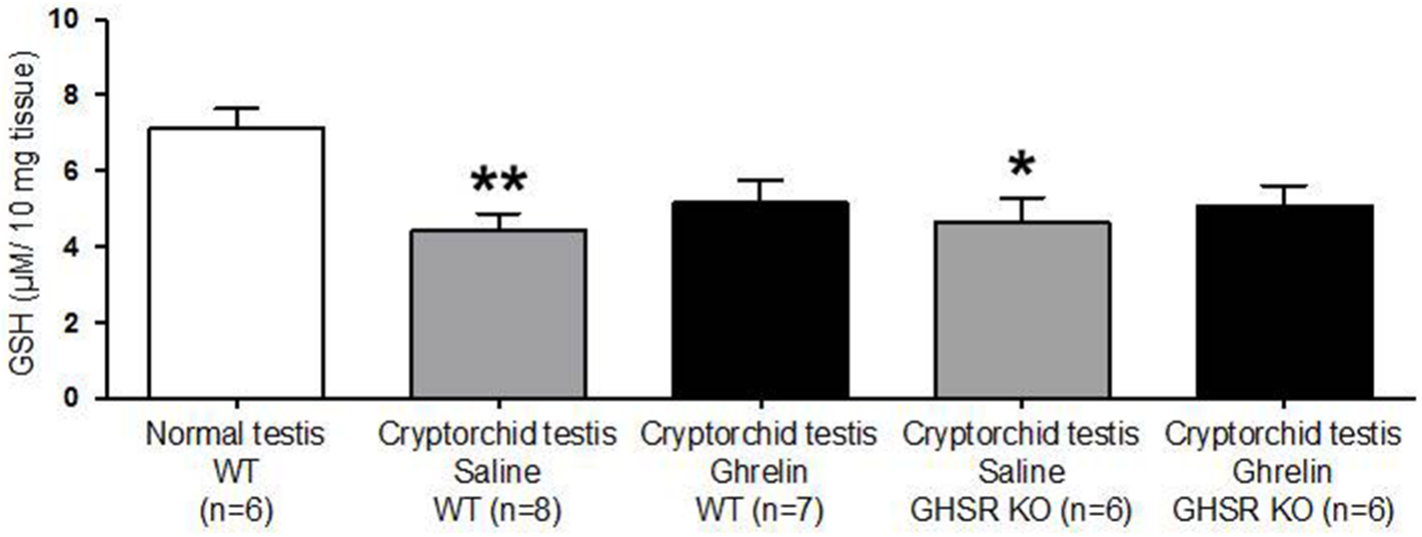
Testicular glutathione (GHS) content after induction of cryptorchidism in wild type and GHSR KO mice. Experimentally-induced cryptorchidism significantly decreased GSH content in WT and GHSR KO mice, while ghrelin treatment ameliorated this effect. All the data were analyzed in comparison to the wild type mouse normal testis by one-way ANOVA multiple comparisons with Dunnett's test and expressed as mean ± SEM. (** p<0.01, * p<0.05).

Changes in glutathione (GSH) content levels in cryptorchid testes of wild type and GHSR KO mice compared to normal testes at 20 days after surgery were evaluated. GSH concentration was significantly decreased in saline-treated cryptorchid wild type (4.430 ± 0.447, n = 8, p-value < 0.01) and GHSR KO (4.646 ± 0.655, n = 6, p-value <0.05) mice compared to normal testes (7.120 ± 0.531, n = 6). Interestingly, ghrelin administration increased GSH concentrations in wild type (5.151 ± 0.606, n = 7) and GHSR KO (5.091 ± 0.530, n = 6) cryptorchid mice decreasing the gap in the GSH levels between cryptorchid and normal testes (Fig 7).

## Discussion

The present study examined the effect of ghrelin on mouse reproductive performance and demonstrated that the protective effects of ghrelin on heat-induced testicular degeneration are not mediated by GHSR-1a receptor. Ghrelin and its functional GH-secretagogue receptor (GHSR) have been detected in rat testes [20]. The testis is an endocrine organ where different cell types cooperate to support germ cells development, under the control of several hormones, growth factors and ghrelin [20, 41]. Previous data established species differences in the testicular localization of ghrelin and its receptor. Ghrelin expression has been detected mainly in Leydig cells of rodents and sheep [20]. Ghrelin is also present in Leydig and Sertoli cells of human and testis but not in germ cells [21]. However, GHSR1a has been located in Leydig and Sertoli cells as well as in germ cells of the human testis [21]. Expression of the functional receptor GHSR-1a has been detected in Sertoli and Leydig cells, but not germ cells, of rat testis [19, 42]. In contrast with human and rodent findings, ghrelin and GHSR1a have been revealed not only in Leydig and Sertoli cells but also in germ cells of adult sheep testis [43]. Recent studies in mouse testis revealed the expression of ghrelin in Leydig cells while GHSR1a has been detected in spermatogenic cells and mature spermatozoa [44, 45]. Moreover, it has been reported that mouse testicular Sertoli cells produce and release ghrelin and express GHSR1a [46].

In this study, we demonstrated that ghrelin and not GHSR1a deletion in mice affect male but not female fertility. To evaluate the reproductive performance of these transgenic mouse lines, female and male heterozygous and homozygous ghrelin knockout mice were paired and the number of pups was measured. Breeding pairs that included ghrelin KO male mice showed a significant decrease in the number of pups (Fig 1A); however, no significant difference in the number of pups for breeding pairs that included GHSR KO mice was detected (Fig 1B). Based on these data we would predict that ghrelin KO mice would display a testicular germ cell hyperplasia and abnormal spermatogenesis. Ghrelin KO mice showed a significant increase in the testicular weights in comparison to the wild type animals (Fig 2A). However, qualitative histopathology of testes did not reveal any significant differences between wild type and both the transgenic mouse lines (Fig 2B, 2C and 2D).

Programmed germ cell death occurs spontaneously during spermatogenesis at a low rate. Normal loss of testicular germ cells is required to limit the number of cells that may be supported by the Sertoli cells [47–49]. Increases in germ cell apoptosis have been observed in specific disease conditions [50, 51], after testicular injury or after exposure to either Sertoli cell or germ cell toxicants [52, 53]. In this study, germ cell apoptosis was assessed in ghrelin KO and GHSR KO normal testes by TUNEL assay and quantified as the percentage of round seminiferous tubules with more than 3 TUNEL-positive cells. A significant higher incidence of seminiferous tubules with more than 3 apoptotic germ cells was observed in ghrelin KO mice but not GHSR KO mice (Fig 3D). Therefore, testicular germ cells spontaneously undergo apoptosis in ghrelin KO testes.

Cryptorchidism is a common congenital abnormality characterized by a failure of descent of one (unilateral) or both (bilateral) testes increasing the risk for developing male infertility, testicular cancer, germ cell loss and impaired spermatogenesis [54–56]. Congenital cryptorchidism occurs in 2–4% of male births and it has been reported that 1–3% of boys develop acquired cryptorchidism during childhood [57, 58]. The temperature of the scrotal testis is 3 degrees Celsius lower than body temperature, and this cooler temperature is essential for spermatogenesis [59]. Many studies have shown that spermatocytes and round spermatids develop DNA damage after heat stress [60, 61], while spermatogonia and elongated spermatids are most resistant to high temperatures [62]. The high abdominal temperature has been reported as a stress factor that induces germ cell apoptosis [63] without damage to the Sertoli cells [64]. However, other studies have suggested that spermatogonia, spermatozoa and Sertoli cells are sensitive to elevated temperatures [65]. As shown in previous studies, surgically induced cryptorchidism caused an initial testicular swelling [66, 67], followed by a reduction in the testicular weight at 4 days post-surgery leading to full testicular atrophy at 20 days post-surgery. The testicular swelling and atrophy were ameliorated by the administration of ghrelin demonstrating that ghrelin treatment causes a delayed response to testicular injury induced by experimental cryptorchidism (Fig 4 and Table 1). The use of GHSR KO cryptorchid mice revealed that the GHSR-1a signal transduction pathway is not required for this ghrelin-mediated protection against testicular injury (Fig 4).

**Table 1:** Cryptorchid/scrotal testis weight ratios at 1, 4 and 20 days post-surgery.

Post-surgery (days)	Saline-treated mice	Ghrelin-treated mice	p-value
**1**	1.134 ± 0.051 (n = 9)	1.019 ± 0.026 (n = 10)	0.0058 **
**4**	0.848 ± 0.040 (n = 8)	0.924 ± 0.044 (n = 8)	ns
**20**	0.300 ± 0.008 (n = 19)	0.352 ± 0.012 (n = 19)	ns

Cryptorchid/scrotal testis weight ratios are reported as the mean ± SEM. Data were analyzed by two-way ANOVA multiple comparisons Fisher’s LSD test

** p < 0.01.

Spermatogenic arrest in experimental cryptorchid mice was associated with the formation of multinuclear giant cells and reduced spermatid number in seminiferous tubules. The present study demonstrated that ghrelin treatment increased the percentage of seminiferous tubules containing spermatids in cryptorchid testes of GHSR KO mice, indicating that this effect was not mediated by GHSR1a receptor but through an unknown pathway. No differences were detected in the seminiferous tubule diameter and in the percentage of seminiferous tubules with multinucleated giant cells in cryptorchid wild type mice between ghrelin and saline groups. On the contrary, ghrelin treatment significantly increased seminiferous tubule diameter in GHSR KO cryptorchid mice testes. The protective effect of ghrelin was enhanced in GHSR KO mice in comparison to wild type mice. One possible explanation is that the absence of the GHSR-1a receptor could activate a compensatory effect, increasing the functional impact of ghrelin. Previous findings reported that this protective effect of ghrelin on the testicular germ cells following surgically induced cryptorchidism seems to be mediated by its antioxidant properties [68]. Since a decrease in glutathione (GSH) content has been previously reported to be an early event in the apoptotic cascade [38, 39], GSH content levels were evaluated in ghrelin- and saline-treated cryptorchid testes of wild type and GHSR KO mice. Our data showed increased GSH content levels after ghrelin exposure in cryptorchid testes of wild type mice compared to the saline-treated group, confirming the antioxidant properties of ghrelin. Furthermore, the increase in GSH content in ghrelin-treated GHSR KO mice demonstrates that the antioxidant properties of ghrelin are not mediated by the GHSR-1a receptor-signaling pathway.

In conclusion, our data have provided direct evidence that the absence of ghrelin has no effect on female reproductive performance, but does compromise male fertility. In addition, ghrelin ameliorated the adverse consequences of surgically induced cryptorchidism, though the GHSR-1a receptor signaling pathway was not required for this protective effect. Therefore, ghrelin may be a viable therapeutic strategy to accelerate the recovery of testicular germ cells following testicular injury.
